# Empowering Cancer Patients: A Scoping Review on Gamified Approaches To Health Literacy for Self-Care

**DOI:** 10.1007/s10916-025-02241-9

**Published:** 2025-09-09

**Authors:** Filipe Cerqueira, Marta Campos Ferreira, Maria Joana Campos, Carla Silvia Fernandes

**Affiliations:** 1 Department of Engineering, FEUP – Faculty of Engineering of the University of Porto, City: Porto, Portugal; 2 Department of Engineering INESC TEC- Institute of Systems and Computer Engineering, Porto, Portugal; 3 Department of Nursing, ESEP – Porto Higher School of Nursing, Rua Dr. António Bernardino de Almeida, nº 830, Porto, 4200-072 Portugal; 4 RISE@health Research Unit, Rua Dr. António Bernardino de Almeida, nº 830, Porto, 4200-072 Portugal; 5 ADITGameS Association, Póvoa de Varzim, Portugal

**Keywords:** Cancer survivors, Gamification, Health education, Oncology patients, Patient empowerment

## Abstract

To address the challenges of self-care in oncology, gamification emerges as an innovative strategy to enhance health literacy and self-care among individuals with oncological disease. This study aims to explore and map how gamification can promote health literacy for self-care of oncological diseases. A scoping review was conducted following the Joanna Briggs Institute guidelines and the PRISMA-ScR Checklist developed for scoping reviews. A comprehensive search strategy was employed across MEDLINE^®^, CINAHL^®^, Scopus^®^, and Web of Science^®^ databases, with keywords focusing on oncological patients and gamification tools applied to self-management, from inception to December 2023. Thirty studies published between 2011 and 2023 were included, with a total of 1,118 reported participants. Most interventions (*n* = 21) focused on the development of mobile applications. The most frequent gamification elements included customizable avatars, rewards, social interaction, quizzes, and personalized feedback. The interventions primarily targeted health literacy and patient education, symptom monitoring, management of side effects, pain control, and adherence to medication and nutrition regimens. The integration of gamification elements into digital health solutions for oncology is expanding and holds promises for supporting health literacy and self-care. Further studies, preferably longitudinal, are needed to assess the effectiveness and impact of these interventions across different oncological populations and clinical settings.

## Introduction

Health literacy is essential for patient education and informed decision-making [1]. Health literacy refers to the ability to obtain, process, and understand health information and services needed to make informed health decisions [1, 2]. In the context of oncology, where the complexities of health information are fundamental to a constant fight against the disease, health literacy takes on added importance [3, 4]. Cancer care demands knowledge of disease, treatment, and self-management [3]. Many cancer survivors are subjected to long periods of treatment, and effective self-care of their health is essential [4]. Active patient involvement improves outcomes and quality of life [3, 4].

However, participation in shared decision-making requires a certain level of communicative health literacy [1, 2]. This set of skills, which involves understanding and using health information effectively, is considered a prerequisite for successful self-care of health. In this sense, information is so necessary that cancer is among the most searched diseases on the Internet [5]. In this review, self-care was considered in a broad sense, encompassing not only how individuals manage their condition but also other crucial aspects. This includes monitoring symptoms, adopting habits that promote well-being, and ensuring that patients have autonomy in decision-making [6].

In the community of cancer survivors, the acquisition of information via the Internet has seen a notable increase [7]. The range of symptoms faced by cancer survivors is diverse, including psychosocial, cognitive, physiological, and even sexual challenges, as well as anxieties related to recurrence and the emergence of new neoplasms [3, 8, 9]. Compared to healthy individuals, cancer patients have more complex health information needs, and inadequate or inaccurate information can result in dissatisfaction or worse outcomes [8].

Self-care implies that patients take an active role in monitoring, understanding and addressing the physical, emotional and social aspects of their health, in collaboration with healthcare professionals [9], where health professionals play an important role. For individuals facing the complexities of oncological diseases, self-care encompasses tasks such as adhering to treatment plans, managing symptoms, adapting lifestyle and managing the psychosocial impact of the disease [10]. It is a dynamic process that requires ongoing education, support and access to resources, encouraging patients to become informed advocates for their health [11].

Just as literacy has consistently supported our efforts in combating disease, technology and its related innovations have become increasingly prominent in recent decades [5, 6]. Within the concept of technology, we cannot fail to mention the term gamification, which has emerged as a captivating approach to the way we interact with technology [12, 13]. Taking advantage of the innate human affinity for play, gamification introduces elements of engagement, motivation and interactive learning into traditionally serious domains [14]. In the health sector, gamification is a promising tool that transcends conventional approaches. By integrating game mechanics into health-related contexts, gamification has the potential to revolutionize the way individuals, particularly those dealing with oncological diseases, engage with and understand information about them [12].

Based on health literacy and the growing field of gamification, the purpose of this scoping review was to explore and map how gamification can promote health literacy for the self-care of oncological disease. To better highlight the importance and impact of this topic, it is crucial to recognize that cancer remains one of the leading causes of morbidity and mortality worldwide, there were approximately 18.1 million new cases and 9.6 million cancer-related deaths globally [5]. In Europe, both the incidence and survival rates of cancer have increased. This creates significant challenges for public health and healthcare systems [5]. Accordingly, this review was guided by the following research question: What evidence is available regarding the use of gamification to promote health literacy for self-care among oncology patients? [5].

## Materials and Methods

### Study Design

A scoping review was conducted following the Joanna Briggs Institute (JBI) guidelines and reported according to the PRISMA-ScR Checklist (Preferred Reporting Items for Systematic Reviews and Meta-Analyses extension for Scoping Reviews) [15]. The study selection process is illustrated in Fig. 2, using the PRISMA 2020 Flow Diagram [16].

The research protocol was registered on the Open Science Framework^®^ platform (OSF 10.17605/OSF.IO/GDY78).

### Search Strategy

A search was conducted in the MEDLINE^®^ (Medical Literature Analysis and Retrieval System Online), CINAHL^®^ (Cumulative Index to Nursing and Allied Health Literature), Scopus^®^, Web of Science^®^. Using the PCC [17] approach to construct clear and meaningful objectives and eligibility criteria for this review the following query was used for the search: *“oncology patient” OR “cancer patient” OR “cancer survivor”) AND KEY=(“gamification” OR “game” OR “video game” OR “app” OR “Digital tool” OR “serious game” OR “mobile application” OR “Web application” OR “Mobile Game” OR “mhealth”) AND KEY=(“self-management” OR “pain management” OR “patient education” OR “adherence” OR “compliance” OR “cancer education” OR “cancer literacy”.*

This query was defined after several tests, trying to use a vast number of different combinations, always bearing in mind the aim of this review. All articles published up to December 2023, were included. Furthermore, the search for additional studies was expanded to involve reviewing the reference lists of all publications chosen for inclusion in the review, a method referred to as “backward citation searching.”

### Eligibility Criteria

The selection included articles describing gamified digital health interventions designed to promote health literacy and self-care among oncology patients and cancer survivors. For the purposes of this review, “gamified technology” was defined as any solution, such as mobile applications, web platforms, or serious games, that incorporated gamification elements into their design, such as points, badges, avatars, challenges, rewards, and interactive feedback, with the aim of increasing user engagement [12]. Included were works published in English, Portuguese, Spanish, or French, focusing on specific technological solutions such as chat-based, telecommunications, and AI solutions. Gamified applications developed primarily for entertainment purposes were excluded. Articles that lacked technological contributions and unrelated surveys were also excluded. All types of primary research articles related to the topic under study were included, regardless of study design. Editorials, commentaries, research protocols, and dissertations were not included. If the full text was not publicly available, corresponding authors were contacted, or the article was obtained through institutional access.

### Data Extraction and Analysis

The PRISMA-ScR Checklist structured the information from the selection process [17]. Rayyan^®^ software facilitated the review’s development. Duplicate records were automatically identified and removed using the Rayyan web application prior to the screening process. The results from these articles were compiled and presented as a narrative summary. A data extraction table was used to systematically organize the relevant studies, cataloging key information from each study. In the last phase of the evaluation, an exhaustive survey of each article was carried out, centred on the extraction of relevant elements. To do this, it was necessary to identify the main dimension addressed (disease self-care or technology), identify the type of focus of the study (education/literacy, symptom monitoring, side effects, pain management, medication/nutrition or gamification) and analyze the determinants and their impact on user perception (negative or positive). As the determinants were perceived and identified, a categorization was carried out, and this system was carefully improved through a repeated process involving the continuous comparison of ideas and concepts based on the literature review. Finally, the categories were integrated and refined to establish comprehensive categories aligned with each dimension. The study selection process, including title and abstract screening as well as full-text review was conducted independently by two reviewers (FC, MJC). Disagreements were resolved through discussion or consultation with a third reviewer (CSF).

## Results

**Fig. 1:**
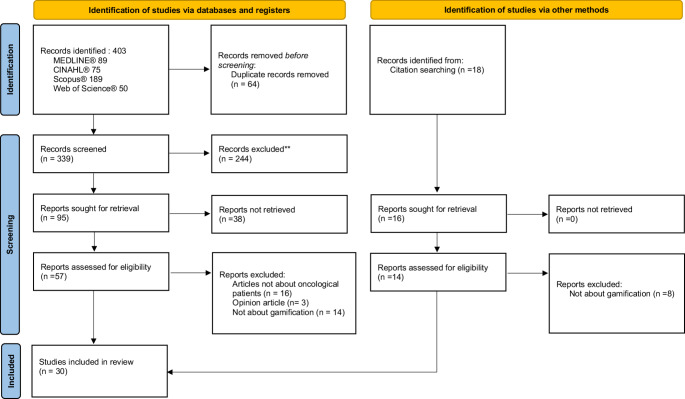
PRISMA flow diagram (2020) [16]

Figure 1 illustrates the process of identifying and including articles, following the PRISMA-ScR^®^ guidelines. Initially, 403 articles were considered for potential inclusion in the study, which 30 articles were included in this review [7, 10, 12, 18–44].

### Characteristics of the Included Studies

Although the search was conducted without time restrictions, 30 studies published between 2011 and 2023 were included. The studies were conducted in various countries, with the highest incidence in the United States (*n* = 9) [12, 23, 31, 35, 36, 38, 39, 44]. Regarding methodology, 18 studies described the development of digital solutions [12, 21, 23–25, 27, 30–37, 39, 41, 42], 10 studies conducted implementation or usability/feasibility trials [12, 18, 20, 31, 32, 35, 37, 38, 40, 43], and 8 studies evaluated the impact on adherence, health literacy, or self-care [12, 23, 25, 31, 35, 38, 39, 44]. It is important to note that several studies incorporated more than one of these objectives (Table 1).

**Table 1:** Characteristics of the included studies

Authors Year Country	Purpose	Study design	Participants	ICT Technology	ICT Solution
Abahussin, West, Wong, Ziegler, Allsop, 2023 [18] Saudi Arabia	Development of an app intended to support pain self-management for patients with cancer.	Pilot study	Not specified	Mobile application	Development of a mobile health app for pain self-management in cancer patients.
Adriaans, Dierick-van Daele, Van Bakel, Nieuwenhuijzen, Teijink, Heesakkers, Van Laarhoven, [19] 2021 Netherlands	Analyze the effectiveness of digital self-management support tools (DSMSTs) on physical and psychosocial symptoms or other supportive care needs in adult patients with cancer.	Review	Not specified	Web-Based Platforms Mobile Apps Gaming	Mobile Health Applications
Ahluwalia, Gupta 2022 [20] India	Development of an integrated pain management app to support pain self-management for cancer patients.	Pilot study	Not specified	Mobile Application	Comprehensive solution for cancer pain management.
Baglione, Tolman, Dapaah, Johnson, Wells, Hall, Barnes [21] 2023 United States	Development of a mobile health app to support symptom and medication management for lung cancer patients.	Experimental study	9 individuals with stage III-IV lung cancer	Mobile Application	Symptom and medication management.
Baranowski, Buday, Thompson, Lyons, Lu, Baranowski [22] 2013 United States	Guidance for investigators considering a Games for Health (G4H) as a behavioral intervention procedure.	Review	Not specified	Game	Behavioral intervention procedure.
Chai, Lau, Tee, Mahmud 2022 [23] Malaysia	Serious game intervention based on the Protection Motivation Theory (PMT) was designed and developed to motivate childhood cancer patients.	Experimental study	24 child-caregiver	Mobile Game	Players take care of a virtual pet’s daily self-care and cancer treatment needs.
Crafoord, Fjell, Sundberg, Nilsson, Langius-Eklöf [24] 2021 Sweden	The study aims to describe engagement with the Interactor app among patients with breast or prostate cancer during treatment.	Experimental study	149 patients	Mobile Application	Developed Interaktor, an interactive smartphone and tablet app, to support patient symptom management
Miranda, Salomé, Costa, Alves 2022 [25] Brazil	Develop and validate a mobile application (app) to educate patients about breast cancer surgical treatment.	Experimental study	13 doctors and 19 breast cancer patients	Mobile Application	Developed the OncoMasto Cirurgia App, after an integrative literature review, testing and validation.
Boulley, Leroy, Bernetière, Paquienseguy, Desfriches‐Doria, Préau 2018 [26] France	This study aims to highlight the components of Digital health interventions (DI), investigate patient engagement with DI, and explore the effects of DI on psychosocial variables.	Review	Not specified	Websites, Mobile Application, smartphone applications, and wearable devices	Not specified
Fraterman, Wollersheim, Tibollo, Glaser, Medlock, Cornet, Gabetta, Gisko, Barkan, Glasspool, Kogan, Lanzola, Leizer, Mallo, Ottaviano, Peleg, Veggiotti, Śniatała, Wilgenhof, 2023 [27] Netherlands	Explore the use of the CAPABLE (Cancer Patients Better Life Experience) app in providing symptom monitoring, education, and well-being interventions.	Pilot Study	Not specified.	Mobile Mobile Application and Smartwatch App	eHealth tool consisting of front end and backend components (mobile app and smartwatch for patients, web-based dashboard for HCPs) that users interact with and that provide functionality related to decision support and coaching.
Gentile, Markham, Eaton 2018 [28] United States	Describe five potential benefits and drawbacks of cancer-related social media and why it is important for oncologists to understand and guide patients to best access social media.	Review	Not specified.	Social Media Platforms	Facebook, Instagram, Twitter and YouTube
Gerling, Fuchslocher, Schmidt, Krämer, Masuch 2011 [29] Germany	Discussed the challenges of evaluating digital games in a hospital setting which were revealed during the evaluation phase.	Experimental study	23 children and juveniles with cancer	Mobile Application	A game concept Cytarius was developed and implemented as a playable prototype. The game is set in a science fiction narrative and integrates different types of cancer and treatment options as game mechanics
Gopichandran, Garg, Chalga, Joshi, Dhandapani, Bhatnagar, 2023 [29] India	Outline the development of a mobile application-based system to develop a physician-patient relationship and to improve adherence to medications prescribed for cancer pain management.	Experimental study	Not specified.	Web and Mobile Application	Application that reminded scheduled medication by alarm, collected medical adherence details, daily symptom observation, and their severity and SOS medication details
Harder, Holroyd, Burkinshaw, Watten, Zammit, Harris, Good, Jenkins 2017 [31] United Kingdom	The study aim was to develop a mobile application (app) supported by user preferences to optimize self-management of arm and shoulder exercises for upper-limb dysfunction (ULD) after breast cancer treatment.	Experimental study	4 breast cancer patients	Mobile Application	bWell, a novel mobile app, was developed together with breast cancer patients to support self-management of arm and shoul-der exercises following axillary treatment.
Jiang, Chen, Yuan, Lin, Chen, Li, Jiang, Yu, Du, Peng 2023 [7] China	Examine the feasibility and preliminary effects of an individualized mHealth nutrition (iNutrition) intervention in post-discharged gastric cancer patients following gastrectomy.	Randomized Controlled Pilot Trial	12 participants	Mobile Application	The iNutrition application is an individually tailored, symptom-directed and theory-based nutritional behavioral management.
Kim, Kim, Shin, Jang, Kim, Han 2018 [12] Republic Of Korea	Evaluate if patient education using a mobile game may increase drug compliance, decrease physical side effects of chemotherapy and improve psychological status in breast cancer patients.	Randomized Controlled Trial	76 patients with metastatic breast cancer	Mobile Application	ILOVEBREAST was developed with an intention to improve self-management and to reduce the side effects of chemotherapy drugs.
Kim, Goldsmith, Sengupta, Mahmood, Powell, Bhatt, Chang, Bhuyan 2019 [13] United States	Evaluate the importance of health literacy and how mobile health application can improve the patient knowledge to better health results	Review	Not specified.	mHealth Apps	Not specified.
Lim, Kim, Yeo, Chae, Yu, Hwang 2023 [32] Republic of Korea	This study investigated the feasibility and usability of a personalized mobile health app for self-management during the year following breast cancer surgery.	Pilot Study	29 patients	Mobile Application.	"Breast Cancer by Second Doctor."was developed for personalized management and self-management of breast cancer patients during the first year following surgery.
Lopez-Perez, Alonso, Filippodou, Mercalli, Cavalieri, Martinelli, Licitra, Manos, María Cabrera-Umpierrez, Fico 2023 [33] Spain and Italy	The objective is to provide a comprehensive strategy for the development of a disease-specific application to monitor Quality of Life in Head and Neck Cancer survivors	Literature Review	Not specified.	Mobile Application	The BD4QoL platform obtained as a result, is made up of a mobile application that collects patient data, and a Point of Care tool where clinicians view and manage patient data.
Masumoto, Nakagami‑Yamaguchi, Nambu, Maeda, Uryu, Hayakawa, Linn, Okamura, Kurihara, Kihira, Deguchi, Hori 2022 [34] Japan	The objective was to developed two age-dependent game-based learning programs, which enable continuous approaches for childhood cancer survivors along their intellectual maturation.	Pilot Study	83 patients.	Tablet or laptop application.	Two game-based learning programs were developed:"FUN QUEST"for school-aged children and"START LINE plus"for adolescents and young adults, both designed to enhance health management awareness among childhood cancer survivors.
Monteiro-Guerra, Ruiz Signorelli, Rivera-Romero, Dorronzoro-Zubiete, Caulfield 2020 [35] Spain, Ireland	Explore insights from breast cancer survivors on motivational and personalization strategies to be used in physical activity coaching apps and interventions.	Pilot Study	24 patients.	Mobile Application.	Not specified.
Oldenmenger, Baan, Van der Rijt 2017 [36] The Netherlands	The purpose of this study was to investigate whether internet monitoring of cancer-related pain in outpatients was feasible.	Pilot Study	84 patients.	Web Application	**Pijn-Kanker**. This web application was developed to monitor cancer-related pain in outpatient settings.
Orlemann, Reljic, Zenker, Meyer, Eskofier, Thiemt, Herrmann, Friedrich Neurath, l Zopf 2018 [37] Germany	This study aimed at investigating the feasibility and applicability of a novel mobile phone app to assess and dietary behaviors in oncologic patients.	Pilot Study	24 patients.	Mobile application.	The goal of the OncoFood app is to manage nutritional documentation, ensuring no entries are forgotten. The app uses acoustic signals to remind users, allowing patients to monitor their current energy and nutrient intake and track their progress.
Peters, Buhrer, Silbernagl, Kayali, Hlavacs, Lawitschka 2019 [38] Austria	The study compared the informative content of health data recorded in a handwritten health diary with that submitted through a serious game.	Pilot Study	11 patients.	Mobile Application.	The core game idea of Interacct is to collect avatars and complete procedurally generated levels. The avatar explores an island and needs to fight off hostile NPC (non-player characters) monsters to complete a level.
Safdari, Ghazisaeidi, Goodini, Mirzaee, Farzi 2016 [39] Iran	The aim of this article was to describe electronic games and their effects on the promotion of behavior change in cancer patients.	Review	Not specified.	Serious Games Interactive Health Games	Not specified.
Salmani, Nahvijou, Sheikhtaheri 2022 [10] Iran	This study was conducted to develop and evaluate the usability of a smartphone-based application for the self-management of patients with colorectal cancer.	Pilot Study	17 patients.	Mobile Application	The ColorectAlong application was developed with features covering several areas of colorectal cancer self-management.
Shalaby, Barakat 2017 [40] Egypt	The objective of the article is to explore the use of"Gamification"in managing the stress of young cancer survivors in a unique and innovative way.	Pilot study	Not specified.	Gamified application	“Mission Possible” that aims to helping those survivors to cope with stress, in an interactive story context, and based on medical questionnaire.
Sin, Kim, Im, Ock, Koh 2023 [41] Republic of Korea	This study aimed to develop a platform (called Smart Cancer Care) to implement a chemotherapy side effect management program and to evaluate its feasibility	Pilot Study	30 patients	Mobile Application	Smart Cancer Care comprises an application for patients and a dashboard for medical staf
Smith, Somers, Kuhn, Laber, Sung, Syrjala, Feger, Kelleher, Majestic, Gebert, LeBlanc,, Owen, Applebaum 2021 [42] United States	The study aims to evaluate the initial response to the"Cancer Distress Coach"mobile app.	Pilot Study	400 patients	Mobile Application	The app provides psychoeducation, symptom tracking, and cognitive-behavioral therapy tools like guided imagery and diaphragmatic breathing, enabling users to manage stress and share progress with clinicians.
Yang, Weng, Chen, Cai, Lin, Hu, Li, Bijuan Lin, Zheng, Zhuang Du, Zheng, Liu 2019 [43] China	The aim was to design, construct, and test the Pain Guard app in patients managing cancer pain	Randomized Controlled Pilot Trial	58 patients	Mobile Application	The Pain Guard app includes two opening screens: one opens to medical staff (the therapeutic interface) and the other opens to patients (the patient interface).
Cosma, Brown, Shopland, Battersby, Seymour-Smith, Matthew, Archer., Masood, A., Khan., A., Graham, Pockley 2016 [44] United Kingdom	The objective is to develop an innovative serious game designed to provide prostate cancer information and risk evaluation specifically tailored for Black African-Caribbean men	Pilot study	29 patients	Serious Game	PROCEE (PROstate Cancer Evaluation and Education), specifically developed for African Caribbean men. It combines elements of prostate cancer education and risk evaluation within a gaming interface

### Characteristics of Participants

The 30 studies included in this review involved a total of 1,118 participants in those studies that reported these data [12, 23, 31, 34, 35, 38–40, 43, 44]. Most participants were adults diagnosed with oncological diseases at different stages of the clinical pathway [12, 23, 31, 35, 38–40, 43, 44]. Some studies specifically targeted children and adolescents with cancer [21, 34, 39, 44], while others included older adults or mixed populations [34, 35, 38–40]. Cancer survivors with different types of malignancies were included. Breast cancer was the most frequent diagnosis among the studies [12, 31, 35, 44], followed by cases of lung cancer [30, 33], head and neck cancer [37], prostate cancer [41], as well as gastrointestinal cancer, melanoma, and leukemia [32, 36, 43]. Pediatric cancer was also represented, with several studies focusing on children and adolescents [21, 34, 39, 44].

### Characteristics of Technology

**Fig. 2:**
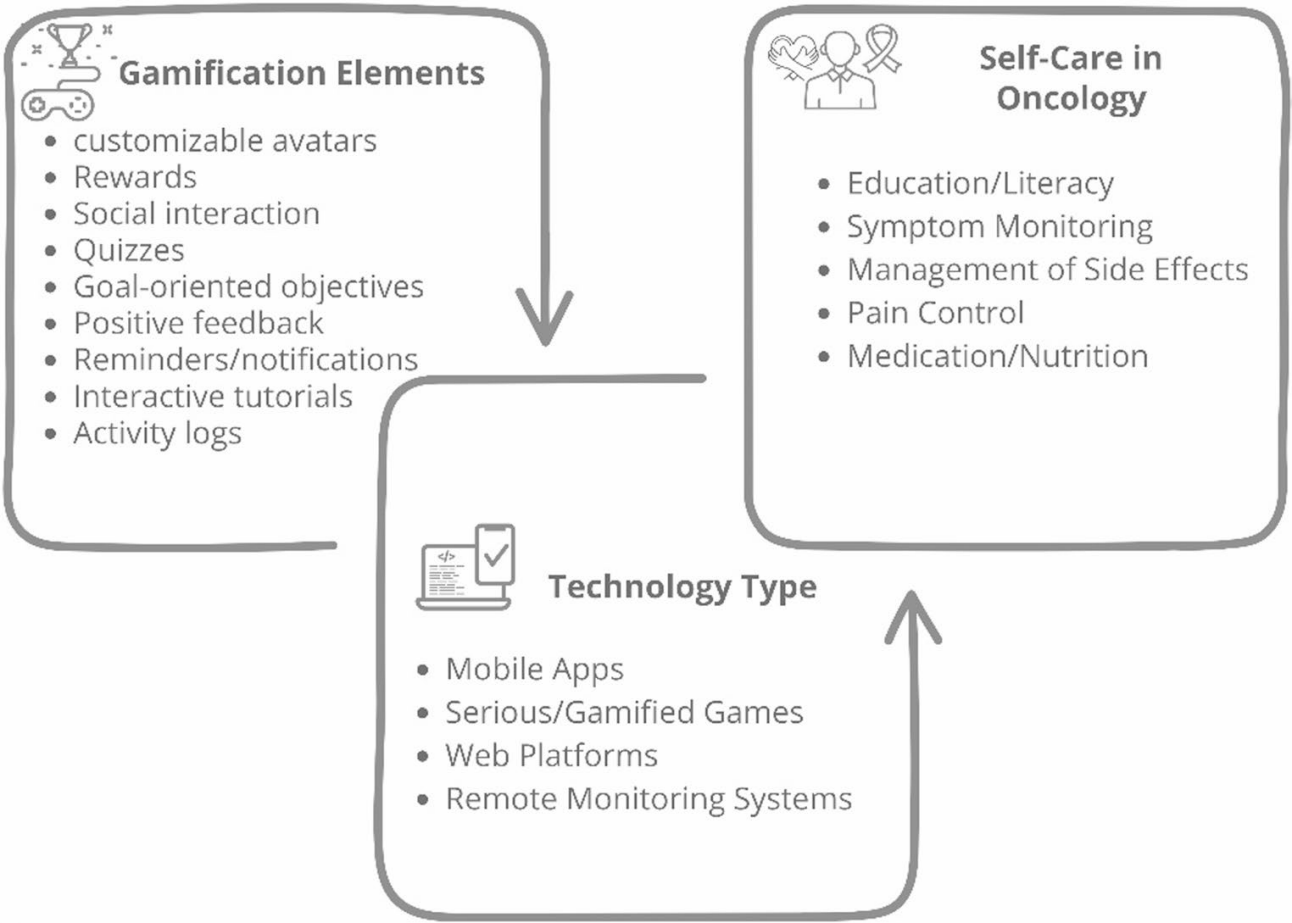
Overview of technology types, main gamification elements, and key self-care domains in digital oncology interventions

Mobile applications were the most frequently referenced resource (*n* = 21) [12, 23, 30–44]. Serious games constituted the second most identified type of resource, being used in six studies [21, 34, 38, 39, 44]. Web platforms appeared in three studies [35–37], generally designed to facilitate remote monitoring, and information sharing between users and healthcare professionals, as well as to provide information aimed at promoting health literacy. Finally, remote monitoring systems were mentioned in two studies [24, 27]. These systems integrated sensors and/or clinical feedback mechanisms to enable real-time monitoring of symptoms or other parameters relevant to disease management. Figure 2 presents the different types of technology identified, the main gamification elements reported in the articles, and the most frequently addressed self-care domains in digital oncology interventions.

### Gamification Elements

The analysis of the studies revealed the use of various gamification elements. Among the most frequent were customizable avatars, which were used in seven studies [12, 23, 34, 35, 38–40]. Rewards, such as virtual prizes, medals, or bonuses, were identified in nine studies [12, 23, 38–40, 44], serving as incentives and maintaining user engagement. Social interaction or participation in virtual communities was reported in five studies [12, 23, 35, 39, 40], allowing for peer support. The integration of simple quizzes was another element identified in four studies [23, 34, 39, 44] to promote learning. The setting of personalized or goal-oriented objectives was also identified (*n* = 5) [23, 35, 39, 40, 44], enabling challenges to be tailored to each user. Positive feedback and the delivery of reminders/notifications were recurrent elements (*n* = 7) [12, 23, 35, 38, 40, 44]. Finally, some studies incorporated interactive tutorials and activity logs [23, 39, 40, 44].

### Self-care in Oncology

The dimension of self-care in oncology encompassed several key categories, namely (Table 2): education/literacy, symptom monitoring, management of side effects, pain control, and medication/nutrition.

**Table 2:** This is mandatory. Please provide

Dimension	Category	Determinants of Negative Perception	Determinants of Positive Perception
Self-management in oncology	Education/Literacy	Adherence Challenges. [31] Difficulty on goal setting (can affect motivation). [31, 44] Hard to get good quality information. [25] Challenges in Digital Literacy. [12] Limited Evidence on Effectiveness. [12, 25] Information Overload. [25] Privacy and Security Considerations. [12]	Visual aids (videos, images). [31] Recovery stages. [31] Progressively more difficult exercises. [31] Reminders (notifications). [31] FAQ sector. [31] High Interest in information. [25] Tailored content. [12] Clear communication. [12] Quizzes. [44] Scenarios. [44] Non-medical interventions can be positive. [32]
	Symptom Monitoring	Technology Accessibility Barriers. [33, 42] Adherence Challenges. [24, 27, 33, 42] Limited Representation of Cancer Types. [24, 42] Challenges in Symptom Grading and Reporting. [24, 27] Privacy and Data Security Concerns. [27, 33] Inconsistent Implementation of Policies. [33] Credibility Concerns. [19] Challenges in Assessing Anxiety and Depression. [19]	Increased Access to Treatment. [19, 24, 42] Tailored Treatment Options. [19, 24, 42] Diverse Treatment Modalities. [42] Integration of Clinician Support. 19, 24, 33, 42] Patient-Centered Care. [27, 42] Improved Patient Education. [27] Remote Monitoring Capabilities. [27, 33] Nonpharmacological Interventions and Well-Being Support. [27] Real-Time Symptom Reporting and Clinical Decision Support. [24, 27] Increases adherence. [24, 27] Potential for Early Detection. [33] Telemedicine and Pandemic Response. [33] Symptom Distress Reduction. [19]
	Side effects	Technological challenges/barriers/biased due to wide range of outcomes. [26, 41] Variables on cultural and background. [26, 41] Heterogeneity and Lack of Standardization. [26] Training and time to learn and adapt to the system. [26]	Patient-Centered Approach. [41] Timely Management. [41] Enhanced Communication. [41] Comprehensive Symptom Management. [41] Tailored Interventions. [26] Psychosocial Support. [26] Promoted continuous usage. [26]
	Pain Management	Lack of Theoretical Foundation/Explanation. [10, 18, 37] Clarification of Design/Usability Concerns. [10, 18, 20] Data Protection/Privacy and Use Policy. [18, 20] Simplification of Wording. [18] Concerns About External Integrations (External links used). [18, 20] Recognition of App Complexity. [18] Concerns About Medication Input. [18, 43] Need for Notifications. [18, 20] Ambiguity in Pain Graphs. Lack of Reason/Motivation to Use the App. [18, 20] Personal (for each patient) pain adjustment. [18, 36, 43] Poor Communication Between Professionals and Patients. [37] Lack of Real-time Monitoring. [43] Lack of Functionality. [10]	Intuitive Design and Navigation. [10, 18, 20] Real-time Consultations and Education. [10, 20, 43] Effectiveness of the System’s Main Functions. [10, 18] Real-Time Monitoring with Personalized Feedback. [18, 36] Recognition of Graphs and Tracking. [18] Effectiveness of Pain Management Functions. [10, 18, 20] Ease of Data Collection. [20] Consideration of User Suggestions. [20] Usability and Accessibility. [10] Reminders and Notes Functionality. [10] Well-Defined and Reasons to use. [18]
	Medication/Nutrition	Challenges in Dietary Record Methods. [37] Limited Resources for Professional Nutritional Counselling. [7, 37] Complexity of the System. [30, 37] Time-Consuming Registration Processes. [30] Technical Complexity. [7, 32] Adherence Challenges. [32] Lack of Regular Reminders. [32] Issues with Data Reporting. [32] Lack of Regular Monitoring. [7] Surveys on user status can be too inconvenient. [21] Battery drains on devices due to running an app. [21] Discreet notifications. [21] Need for minimal disruption. [21] Limited Mention of Data Security Measures. [10] Security and Privacy Concerns. [10, 30]	Individualized Nutritional Intervention. [7, 32, 37] Motivational Features. [7, 37] Nutritional Status. [7, 37] User-Friendly Interface. [30, 32] Remote Monitoring and Synchronization. [30] Security and Privacy Measures. [10, 30] Alarm and Medication Management. [30] Healthcare Professional Assistance. [32] Usability and User Engagement. [7, 32] Notifications customized. [21] Medication reminder. [10] Multilingual Support. [10]

#### Education/Literacy

Within the self-care dimension, it is possible to observe how tools and strategies can empower users to effectively manage their health conditions. The objective of technological resources in this category was to promote health literacy among patients by facilitating access to information about the disease and its treatment [12, 23, 25, 31, 34, 35, 39, 40, 44]. On the positive side, the use of visual resources such as videos and images, together with personalized content and clear communication, enhances the learning experience and ensures greater adherence to educational programs. However, challenges such as information overload [25], difficulty accessing high-quality information [25], limited digital literacy [12], and privacy concerns [12] were frequently reported.

#### Symptom Monitoring

In this area, the primary focus was on simplifying symptom monitoring by patients, with the aim of promoting self-control and self-care of disease and treatment-related signs and symptoms. Reported advantages (*n* = 8) [12, 23, 27, 31, 35, 37, 39, 44] included simplified access to care, tailored solutions, proximity to healthcare professionals, and remote support. Disadvantages included technological barriers [33, 42], adherence difficulties [24, 27, 33, 42], limited representation of some cancer types [24, 42], and data privacy concerns [27, 33].

#### Side Effects

In this category, the goal of technological resources was to control the side effects of cancer treatment, ensuring timely response. The studies[26, 41] identified benefits such as patient-centered approaches, timely management, improved communication, personalized interventions, psychosocial support, and promotion of continuous use. On the other hand, technological and cultural barriers, lack of standardization, and the need for training were highlighted as significant obstacles [26, 41].

#### Pain Management

Resources in this category aim to facilitate pain management by providing tools to support pain monitoring and control. In six studies [10, 12, 18, 20, 31, 43], several advantages were reported, such as user-friendly interfaces, tailored responses, and immediate contact with healthcare professionals. Among the disadvantages were the lack of theoretical basis in some resources [10, 18, 20], concerns regarding data protection, and difficulties in integrating with other systems.

#### Medication/Nutrition

In the medication/nutrition category, the objective was to assist patients in managing their therapeutic and dietary regimens, encouraging personalized and motivating approaches. The studies [7, 30, 32, 37, 44] demonstrated the importance of user-friendly platforms, remote monitoring, and customized alerts, in addition to tailored dietary plans for each patient. Major challenges included system complexity, lengthy processes, technical issues (e.g., battery consumption), and lack of regular notifications [21, 30, 32, 37].

## Discussion

The purpose of this scoping review was to identify and gather information on how gamification can enhance health knowledge, aiming at self-care among oncology patients. This was a novel field; despite the significant increase in the use of digital technologies in healthcare, the specific application of gamification elements for individuals with cancer was recent [45, 46], bringing unique challenges both in the structuring of interventions and in their execution and adherence by patients. Although the literature search was conducted without temporal Limits, only articles published from 2011 onwards were identified.

Although no formal assessment of methodological quality or risk of bias was performed, it should be noted that most of the included studies were research articles focused on technological development. Eighteen studies described the development of digital solutions [21, 23–27, 30–37, 39, 41, 42], and ten studies conducted implementation or usability/feasibility trials [12, 18, 20, 31, 32, 35, 37, 38, 40, 43]. Additionally, several studies had relatively small sample sizes and very short intervention periods, which may limit the generalizability of their findings. In summary, most studies focused primarily on the development and preliminary evaluation of the resources, often lacking robust medium- or long-term impact measures or rigorous control groups.

This review highlights that gamification has been proposed as a strategy to address the specific challenges of cancer care, with the aim of increasing patient engagement, autonomy, and health literacy [12, 23, 34, 39]. Mobile applications were most cited; other solutions included games, websites, and monitoring systems [12, 23, 30–44]. Other technologies were referenced, such as educational games [21, 34, 38, 39, 44], websites [35–37], and remote monitoring systems [24, 27]. Notably, breast cancer was the most represented diagnosis, which, while in line with global epidemiological data and the importance of health education in breast cancer care, highlights the need to expand and adapt these solutions to other cancer types, such as lung, prostate, and childhood cancers [12, 24, 25]. This concentration in a specific population demonstrates the need to broaden the scope to other groups.

Regarding gamification elements, the included studies reported the use of features designed to motivate, sustain interest, promote health literacy, and facilitate self-care among cancer patients. The most common elements included customizable avatars [12, 23, 34, 35], rewards such as virtual prizes, medals, or bonuses [12, 23, 38, 39], and social interaction or participation in online communities [12, 23, 35]. These resources were designed to foster a sense of achievement and social support, which are considered important for patients undergoing prolonged and complex treatments. Simple quizzes [23, 34, 39] and clear goals [23, 35, 40, 44] were also frequently used, supporting learning and habit formation. Other important elements included positive feedback, reminders/notifications [12, 23, 35, 38] and interactive tutorials/activity logs [23, 39, 40, 44].

The use of these elements is particularly relevant for oncology patients, who often require continuous support for symptom management, adherence, and information retention [11, 12]. Gamification has been used with the intention of making learning more engaging and supporting self-care by providing clear pathways and motivation for consistent health behaviors [48, 49]. It is important to note that literature increasingly recognizes the potential of games in healthcare contexts. Reviews of the use of games in palliative care [45] and the integration of card games with patients [46] highlighted that game-based approaches could facilitate communication, emotional support, and engagement, even in highly complex clinical settings such as oncology and palliative care [45, 46, 49].

As for the main objectives of technological resources to promote health literacy and self-care among oncology patients, the key objectives identified in the analyzed studies included: promoting health literacy [12, 23, 34, 35], facilitating symptom monitoring [12, 23, 37, 39, 44], management of side effects [26, 41], supporting pain management [10, 12, 18], and increasing adherence to medication and nutrition regimens [30, 32, 37]. One of the main focuses was education, aiming to increase literacy and empower patients for self-care [12, 23, 25, 31, 34, 35, 39, 40, 44]. However, to be effective, these tools must provide reliable, clear, and actionable information [24, 25, 42]. Symptom control was another focus, with several studies showing the use of apps with gamification elements to help monitor symptoms and side effects, supporting remote management, facilitated rapid intervention and empowered patients [12, 23, 27, 31, 35]. Nevertheless, challenges remain regarding the accuracy and accessibility of these tools for symptom classification and the effort required from patients to enter and use data [27, 38]. The reviewed articles emphasized the need for intuitive design and real-time feedback, but also warned of the risk of cognitive overload and the importance of linguistic and cultural adaptation of interventions [18]. Recent studies have also highlighted the challenge of information overload among oncology patients, which can negatively affect engagement and comprehension [25, 28]. Some studies reported the challenge of balancing the amount of information provided to users, aiming to support decision-making without causing information overload [25, 28, 46–48].

Based on the results of this review, several practical recommendations can be made for clinicians, developers, and researchers interested in this topic. Clinicians should begin integrating other types of resources into clinical practice to promote their patients’ health literacy, prioritizing digital tools and gamified interventions whose content has scientific validation. Developers should adopt participatory and user-centered design strategies, involving patients and healthcare professionals throughout the process to increase engagement, motivation, accessibility, and, above all, the validity of the content integrated into these resources. For researchers, future studies should increase their scope to include different types of participants, pathologies, and age groups, and focus on the medium- and long-term effectiveness and the real-world impact of these interventions.

### Limitations and Future Research Directions

This review presents some methodological limitations, such as the possible omission of unpublished studies, grey literature, and articles in languages other than English, Portuguese, Spanish, and French. Most of the included research focused on breast cancer, which restricts the applicability of the findings to other cancer types. The rapid evolution of digital technologies may also cause some findings to become quickly outdated.

Regarding the mapping of gamification elements, this review only identified their presence without evaluating their clinical effectiveness or patient outcomes. On the other hand, the limitations reported by the authors of the analyzed articles highlight potential usability barriers related to low digital literacy, cognitive difficulties, information overload, and the need for cultural or linguistic adaptation. Furthermore, many resources lacked rigorous scientific validation and regular content updates, which may affect their credibility and sustainability. Therefore it is recommended that future research employ participatory and user-centered methods to deepen the understanding of the diverse user experience. At the same time, it will be crucial to validate these resources through longitudinal research.

## Conclusion

In conclusion, this scoping review explored and mapped how gamification has been incorporated into digital technologies aimed at promoting health literacy for self-care among oncology patients. Most of the technological resources were mobile applications, and the most common gamification elements included customizable avatars, rewards, quizzes, and goal-based objectives.

However, some challenges remain, related to the need for scientific validation of content, cultural adaptation, and user-centered design, especially for populations with lower digital literacy or in diverse clinical settings. Further research is needed to evaluate the effectiveness, acceptance, and impact of gamified interventions in different oncology populations and clinical contexts, particularly through longitudinal studies.

## Data Availability

No datasets were generated or analysed during the current study.
